# Crawling Motility on the Host Tissue Surfaces Is Associated With the Pathogenicity of the Zoonotic Spirochete *Leptospira*

**DOI:** 10.3389/fmicb.2020.01886

**Published:** 2020-08-05

**Authors:** Jun Xu, Nobuo Koizumi, Shuichi Nakamura

**Affiliations:** ^1^Department of Animal Microbiology, Graduate School of Agricultural Science, Tohoku University, Sendai, Japan; ^2^Department of Bacteriology I, National Institute of Infectious Diseases, Tokyo, Japan; ^3^Department of Applied Physics, Graduate School of Engineering, Tohoku University, Sendai, Japan

**Keywords:** bacterial motility, pathogenicity, spirochete, leptospirosis, zoonosis, host–pathogen association, biophysics, optical microscopy

## Abstract

Bacterial motility is crucial for many pathogenic species in the process of invasion and/or dissemination. The spirochete bacteria *Leptospira* spp. cause symptoms, such as hemorrhage, jaundice, and nephritis, in diverse mammals including humans. Although loss-of-motility attenuate the spirochete’s virulence, the mechanism of the motility-dependent pathogenicity is unknown. Here, focusing on that *Leptospira* spp. swim in liquid and crawl on solid surfaces, we investigated the spirochetal dynamics on the host tissues by infecting cultured kidney cells from various species with pathogenic and non-pathogenic leptospires. We found that, in the case of the pathogenic leptospires, a larger fraction of bacteria attached to the host cells and persistently traveled long distances using the crawling mechanism. Our results associate the kinetics and kinematic features of the spirochetal pathogens with their virulence.

## Introduction

Many bacteria utilize motility to explore environments for survival and prosperity. For some pathogenic species, the motility is a virulence factor (Josenhans and Suerbaum, 2002). For example, *Helicobacter pylori* requires motility to migrate toward the epithelial tissue in the stomach (Clyne et al., 2000), and motility and chemotaxis are key factors that guide host invasion in different *Salmonella* serovars (Siitonen and Nurminen, 1992; Olsen et al., 2013). Movement and adhesion of the Lyme disease spirochete *Borrelia burgdorferi* in blood vessels are thought to be important during the process of host cell invasion (Ebady et al., 2016). In the enteric pathogens, such as the enteropathogenic *Escherichia coli* (EPEC), the motility machinery flagella are also important for adhesion to the host intestinal epithelium (Cheney et al., 1980).

In this study, we address the association of bacterial motility with pathogenicity in the worldwide zoonosis leptospirosis. The causative agent of leptospirosis *Leptospira* spp. are Gram-negative bacteria belonging to the phylum Spirochaetes. The genus *Leptospira* is comprised of two major clades, “Saprophytes (S)” and “Pathogens (P)” and is further divided into four subclades called P1, P2, S1, and S2 (Vincent et al., 2019). *Leptospira* spp. are classified into over 300 serovars defined based on the structural diversity of lipopolysaccharide (LPS) (Cerqueira and Picardeau, 2009; Picardeau, 2017). The pathogenic species affect various mammalian hosts such as livestock (cattle, pigs, horses, and others), companion animals (dogs and others), and humans, causing severe symptoms, such as hemorrhage, jaundice, and nephritis in some host-serovar pairs (Bharti et al., 2003; Adler and de la Peña Moctezuma, 2010; Picardeau, 2017). The leptospires can be maintained in the renal tubules of recovered animals or reservoir hosts, and the urinary shedding of leptospires to the environment leads to infection in humans and other animals through contact with contaminated soil or water. Although the pathogenic mechanism of leptospirosis is not well elucidated, their motility is known to be somehow involved in pathogenicity using loss-of-motility mutants in animal models (Lambert et al., 2012; Wunder et al., 2016).

**FIGURE 1:**
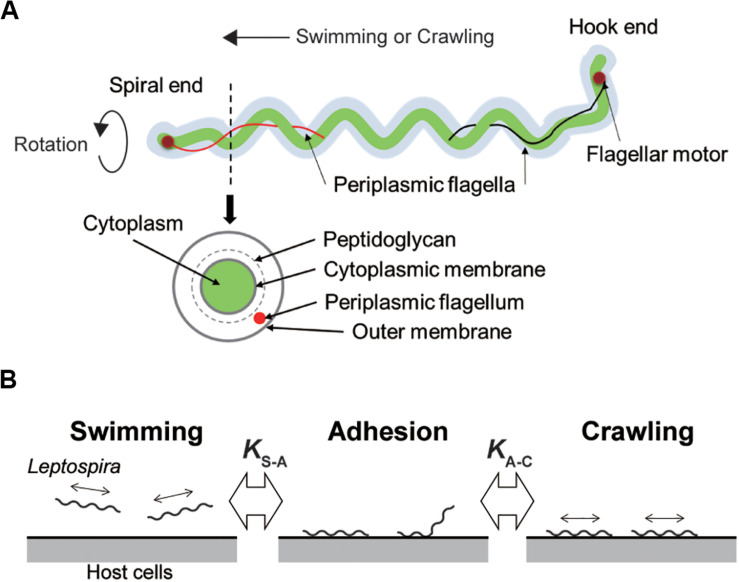
Structure of *Leptospira* and working model. **(A)** Schematic diagram of the *Leptospira* cell structure. **(B)** A three-state kinetic model assuming a transition between “swimming” (floating above cell layers without physical contact to the cells) and “adhesion” (attachment to the cell layer without migration), and between “adhesion” and “crawling” (attachment to the cell layer and movement over surfaces), with *K*_S–A_ and *K*_A–C_ represent the equilibrium constants of each transition, respectively.

*Leptospira* spp. possess two periplasmic flagella (PFs) beneath the outer cell membrane (Figure 1A). The rotation of the PFs gyrates both ends of the cell body and rotates the coiled cell body (protoplasmic cylinder), allowing the spirochete to swim in fluids (Goldstein and Charon, 1990; Nakamura et al., 2014). *Leptospira* cells have high adhesivity to solid surfaces and show diverse behaviors over surfaces: some of the adhered leptospires stay at the same position (so-called adhesion state), whereas the others retain mobility on surfaces (Cox and Twigg, 1974). The surface motility designated as “crawling” is a slip-less movement given by rotation of the protoplasmic cylinder and unidentified adhesins that are freely move on the outer membrane (Tahara et al., 2018). Adherence and entry of pathogenic leptospires in the conjunctival epithelium (Bharti et al., 2003) and in the paracellular routes of hepatocytes (Miyahara et al., 2014) were previously observed using scanning electron microscopy, suggesting adhesion to the host tissue surfaces and subsequent crawling of pathogenic leptospires. To verify this hypothesis, assuming the transition of leptospires between swimming and adhesion states and between adhesion and crawling states in the equilibrium (Figure 1B), we investigated the adhesion and crawling motility of *Leptospira* on the cultured kidney cells of various mammalian species.

## Materials and Methods

### *Leptospira* Strains and Growth Conditions

Pathogenic serovars of *L. interrogans* serovar Icterohaemorrhagiae (strain WFA135), an isolate from *Rattus norvegicus* in Tokyo, Japan, serovar Manilae (strain UP-MMC-NIID) (Koizumi and Watanabe, 2004; Fujita et al., 2015; Tomizawa et al., 2017) and a saprophytic *L. biflexa* serovar Patoc (strain Patoc I) were used in this study. The serovar of WFA135 was determined by multiple loci variable number of tandem repeats analysis (MLVA) and DNA sequencing of the *lic12008* gene (Koizumi et al., 2015; Santos et al., 2018). Bacteria were cultured in enriched Ellinghausen-McCullough-Johnson-Harris (EMJH) liquid medium (BD Difco, NJ, United States) containing 25 μg/mL spectinomycin at 30°C for 2 (*L. biflexa*) or 4 (*L. interrogans*) days until log phase. To track *Leptospira* cells when in co-culture with mammalian kidney cells, a green fluorescent protein (GFP) was constitutively expressed in each strain (Supplementary Movie S1).

### Mammalian Cells and Media

Mammalian kidney epithelial cell lines used included MDCK-NBL2 (dog), NRK-52E (rat), Vero (monkey), MDBK-NBL1 (cow), TCMK-1 (mouse), and HK-2 (human). MDCK, Vero, TCMK, and MDBK cells were maintained in Eagle’s minimum essential medium (MEM) (Sigma-Aldrich, Darmstadt, Germany) containing 10% fetal bovine serum or 10% horse serum (Nacalai Tesque, Kyoto, Japan) at 37°C and 5% CO_2_. NRK cells were maintained in Dulbecco’s modified Eagle’s medium (DMEM) (Thermo Fisher Scientific, MA, United States) with 4 mM L-glutamine, adjusted to contain 1.5 g/L sodium bicarbonate and 4.5 g/L glucose, and with 5% bovine calf serum (Nacalai Tesque) at 37°C and 5% CO_2_. HK-2 cells were maintained in keratinocyte serum-free (KSF) medium with 0.05 mg/mL bovine pituitary extract and 5 ng/mL human recombinant epidermal growth factor (Gibco – Thermo Fisher Scientific, Waltham, MA, United States). All culture conditions contained a 5% antibiotic/antimycotic mixed solution (Nacalai Tesque). Cells were treated with a 0.1% trypsin – EDTA solution (Nacalai Tesque) to dislodge the cells during each passage process.

### GFP Expression in *L. interrogans* and *L. biflexa*

For the construction of a replicable plasmid in *L. interrogans*, the corresponding *rep-parB-parA* region of the plasmid pGui1 from the *L. interrogans* serovar Canicola strain Gui44 plasmid pGui1 (Zhu et al., 2014) was amplified from a *L. interrogans* serogroup Canicola isolate, and the amplified product was cloned into the PCR-generated pCjSpLe94 (Picardeau, 2008) by NEBuilder HiFi DNA Assembly cloning (New England BioLabs), generating pNKLiG1. The *flgB* promoter region (Bono et al., 2000; Bauby et al., 2003) and *gfp* were amplified from pCjSpLe94 and pAcGFP1 (Clontech), respectively, and the amplified products were cloned into the *Sal*I-digested pNKLiG1 for *L. interrogans* or the *Sal*I-digested pCjSpLe94 for *L. biflexa*. The plasmids were transformed into strains WFA135, UP-MMC-NIID or Patoc I by conjugation with *E. coli*β2163 harboring the plasmid (Slamti and Picardeau, 2012). We used the *Leptospira* strains stored at –80°C when created the GFP-expressing transformant. After the strains were recovered from –80°C, we passaged them once or twice prior to the conjugation experiment. The colonies of the transformant were cultured in EMJH and passaged twice, and then log phase cultures were stored at −80°C. The GFP-expressing strains were passaged less than four times for the motility assays, that is, less than eight times of passage after recovered from −80°C for the experiments. Primer sequences used in this study are listed in Supplementary Table S1. Expression of GFP did not affect motility in the *Leptospira* serovars (Supplementary Figure S1).

### Preparation of Kidney Cells and *Leptospira* Cells in a Chamber Slide

Kidney cells were harvested with 0.1% trypsin and 0.02% EDTA in a balanced salt solution (Nacalai Tesque) and plated onto a chamber slide (Iwaki, Tokyo, Japan) using their corresponding media without antibiotics. The slides were incubated for 48 h until a monolayer was formed and washed twice with media to remove non-adherent cells. The cells were incubated for a further 2 h at 37°C and 5% CO_2_. Approximately 500 μL of stationary phase *Leptospira* cells were harvested by centrifugation at 1,000 × *g* for 10 min at room temperature, washed twice in PBS, then resuspended in the corresponding kidney cell culture media without antibiotics at 37°C to a concentration of 10^7^ cells/mL. These suspensions (1 mL) were then added into the corresponding chamber slides containing the kidney cell layer, and the chamber slides were incubated at 37°C for 1 h.

### Microscopy Observation and Adhesion-Crawling Assay

**FIGURE 2:**
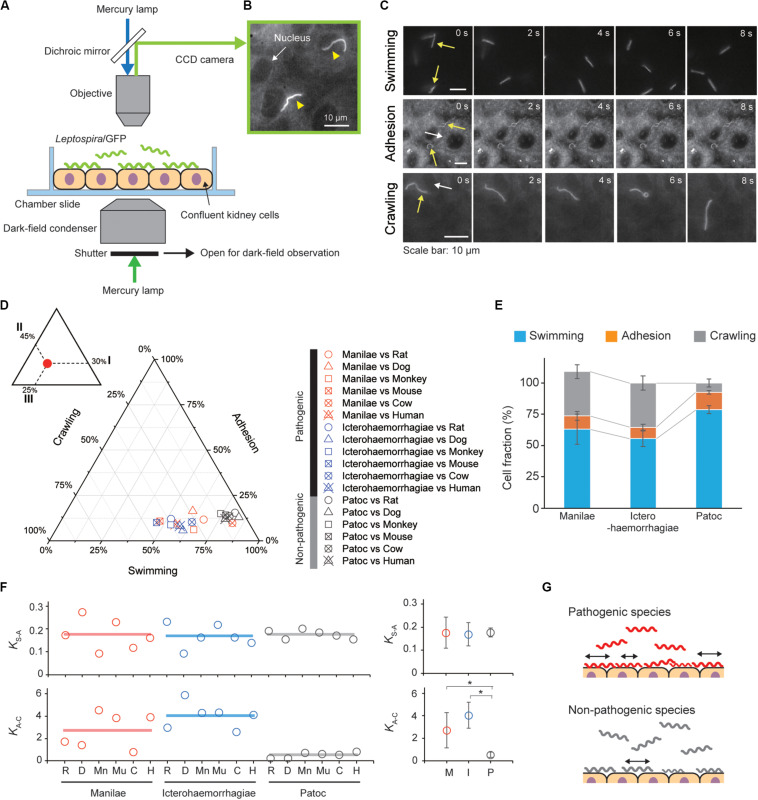
Steady-state motility and adhesion of *Leptospira* cells on kidney cells. **(A)** Schematic of the motility assay on the cultured kidney cells by epi-fluorescent microscopy. Fully confluent kidney cells from either animals or humans were cultured in an observation chamber, and *Leptospira* cells that constitutively expressed GFP were added to the cultures. **(B)** Example of an epi-fluorescence image of the *L. interrogans* serovar Icterohaemorrhagiae, as indicated by the yellow triangles, on the rat kidney cell line, NRK-52E. The nucleus of the kidney cell is shown by the white arrow (Supplementary Movie S1). **(C)** Image sequences of swimming (top), adhered (middle), and crawling (bottom) leptospiral cells. Yellow and white arrows indicate *Leptospira* cells and kidney cells, respectively. No kidney cells were observed due to out of focus during the measurement of *Leptospira* cells swimming in liquid media. **(D)** Ternary plot of the cell fractions in a state of swimming, adhesion, or crawling. The inset schematically explains how to read the ternary plot using an example plot with 30% for I, 45% for II, and 25% for III. Legend is shown to the right of the ternary plot, and M, I, and P indicate the *L. interrogans* serovar Manilae, *L. interrogans* serovar Icterohaemorrhagiae, and *L. biflexa* serovar Patoc, respectively. Each data point represents the mean of triplicate experiments and ∼ 2,400 leptospiral cells were measured per host-serovar pair. **(E)** Average values of the cell fractions. Error bars are the standard deviation. **(F)** The host–bacterium dependence of the equilibrium constants *K*_*S–A*_ and *K*_*A–C*_, calculated from the data shown in panel **(D)**; rat (R), dog (D), monkey (Mn), mouse (Mu), cow (C), and human (H). The averaged values determined for each bacterial strain are shown by horizontal lines (left) and are plotted with the standard deviation (right); *L. interrogans* Manilae (M), *L. interrogans* Icterohaemorrhagiae (I), and *L. biflexa* Patoc I (P). The statistical analysis was performed by Mann–Whitney *U* test (*P < 0.05). **(G)** Schematic explanation of the kinetic difference between pathogenic and non-pathogenic leptospires.

The movement behaviors, swimming, adhesion and crawling of the *Leptospira* cells on the kidney cells were observed using a dark-field microscope (BX53, Splan 40×, NA 0.75, Olympus, Tokyo, Japan) with an epi-fluorescent system (U-FBNA narrow filter, Olympus) and recorded by a CCD-camera (WAT- 910HX, Watec Co., Yamagata, Japan) at 30 frames per second. *Leptospira* cells were tracked using an ImageJ (NIH, MD, United States)-based tracking system and the motion parameters such as motile fraction, velocity and the mean square displacement (MSD) were analyzed using Excel-based VBA (Microsoft, WA, United States). The two-dimensional MSD of individual leptospiral cells during a period Δ*t* was calculated by the following equation: *MSD*(Δ*t*) = (*x*_*i* + Δ*t*_−*x*_*i*_)^2^ + (*y*_*i* + Δ*t*_−*y*_*i*_)^2^, where (*x*_*i*_, *y*_*i*_) is the bacterial position at *I* (see also Supplementary Figure S2). Swimming cells were distinguished from adhesion or crawling cells by the difference in a focus (see Figure 2C), and adhesion was discriminated from crawling based on criteria of both crawling speed (<1 μm/s) and MSD slope (<0.5).

## Results

### Steady-State Analysis of *Leptospira* on the Kidney Cells

We infected cultured kidney cells from six different host species (rat, dog, monkey, mouse, cow, and human) with three *Leptospira* strains (the pathogenic *L. interrogans* serovars Icterohaemorrhagiae and Manilae, and the non-pathogenic *L. biflexa* serovar Patoc) expressing GFP within a chamber slide (Figure 2A). We observed the *Leptospira* cells by epi-fluorescent microscopy (Figure 2B) and measured the fractions of swimming [S], adhered [A], and crawling [C] bacteria on the kidney cells (Figure 2C). Figures 2D,E show that almost half of the pathogenic population transited from the swimming state to the adhesion and crawling states, whereas >75% non-pathogenic leptospires remained in the swimming state. We calculated the equilibrium constant between swimming and adhesion states (*K*_*S*–*A*_) and between adhesion and crawling states (*K*_*A*–*C*_) from the cell fractions using *K*_*S*–*A*_ = [A]/[S] and *K*_*A*–*C*_ = [C]/[A], respectively. The pathogenic leptospires had a significantly larger *K*_*A–C*_ in comparison with the non-pathogenic strain (*P* < 0.05); *K*_*S*–*A*_ did not seem to correlate with virulence (Figure 2F). These thermodynamic parameters suggest that the biased transition from adhesion to crawling would be responsible for the virulence of *Leptospira* (Figure 2G).

### Crawling Motility

**FIGURE 3:**
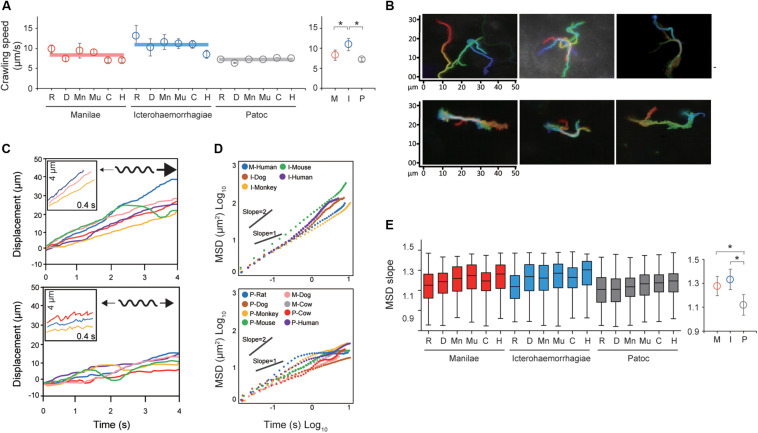
Analysis of the crawling movement of *Leptospira* on kidney cell layers. **(A)** Average crawling speeds determined for each host–bacterium pair. More than 90 bacteria were measured for each pair. The averaged values calculated for each bacterial strain are shown by horizontal lines (left) and are plotted with the standard deviation (right). **(B)** Examples of persistent crawling (upper panels) and diffusive crawling (lower panels) of *Leptospira* cells obtained by single-cell tracking in 10 s. Colors indicate time courses in the order of red orange yellow green blue, and indigo. **(C)** Example time courses of leptospires crawling on the kidney cell surfaces; inset, expanded view of cell displacements and schematics of motion patterns. The upper and lower panels show the long-distance migration represented by directive crawling and the limited migration due to frequent reversal, respectively. **(D)** Examples of MSD vs. time plots, evaluating the directivity of individual leptospiral cell movements: Plots with slopes ∼2 indicate persistently directive crawling (upper), whereas those with ∼1 indicate a non-directional movement (lower), i.e., motion with high frequency of reversal patterns and short net migration distance [refer to the schematic explanation of motion pattern in insets of panel **(C)**]. **(E)** MSD slopes determined for each host–bacterium pair. The boxes show the 25th (the bottom line), 50th (middle), and 75th (top) percentiles, and the vertical bars show the minimum and maximum values. The right panel show the strain-dependence of the medians. The statistical analysis was performed by Mann–Whitney *U* test (*P < 0.05).

Taking the results of the steady-state analysis, we focused on the crawling motility of individual *Leptospira* cells on the kidney cells. Although the crawling speed varied among the measured host–bacterium pairs, *L. interrogans* serovar Icterohaemorrhagiae showed significantly faster speed than the others, indicating the species/serovar dependence of the crawling ability (Figure 3A). On the other hand, there is no difference in crawling speed between *L. interrogans* serovar Manilae and *L. biflexa* serovar Patoc, suggesting that the crawling speed itself is not related to leptospiral virulence. Meanwhile, we observed that some leptospiral cells attached to the kidney cells and moved smoothly for periods, migrating over long-distances (upper panels of Figures 3B,C and Supplementary Movie S2), whereas others frequently reversed the crawling direction (lower panels of Figures 3B,C and Supplementary Movie S3). Cell movements with reversals are considered to be diffusive and thus can be evaluated by plotting MSD against time, a general methodology for diffusion (Brownian motion) analysis (Kusumi et al., 1993). The MSD of simple diffusion without directivity is proportional to time, and therefore double-logarithmic MSD plots from such non-directional diffusion represent slopes of ∼1, whereas those from directive movements show MSD slopes of ∼2, representing the relatively long distance traveled by the cells (Supplementary Figure S2). Double-logarithmic MSD plots obtained from each individual leptospires showed a wide range of MSD slopes (example data are shown in Figure 3D) and differed for each host–*Leptospira* pair (Figure 3E left and Supplementary Figure S3). The non-pathogenic strain showed the slope of ∼1, while the pathogenic strains had significantly larger slopes that denote directive motion (Figure 3E, right). Thus, concerning the crawling motility, directivity and persistency rather than speed could be crucial for virulence.

## Discussion

**FIGURE 4:**
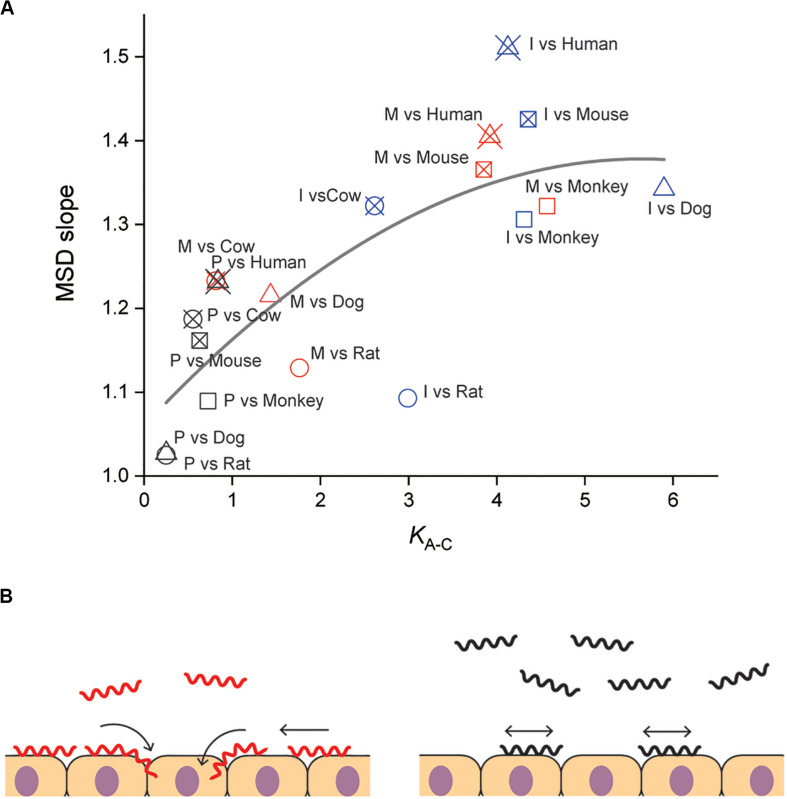
Correlation of adhesion, motility and pathogenicity. **(A)** Relationship between *K*_A–C_ and MSD slope (i.e., crawling persistency). The gray line is the result of quadratic curve fitting. The correlation coefficient is 0.77. See Figure 2D for symbols. **(B)** A plausible model for crawling-dependent pathogenicity of *Leptospira*. In the cases that lead to severe symptoms (left), leptospiral cells were biased to the crawling state, and most of the crawling cells showed directional translation persistently over host tissue surfaces, increasing the invasion probability. For the asymptomatic or non-infectious cases, many leptospires remained in the swimming state and might be removed through body fluids or urination. Some fractions of the adhered leptospires were able to crawl, but their migration distances were limited due to frequent reversal (right).

Our results suggested the importance of adhesion to and persistent crawling on the host tissue for the pathogenicity of *Leptospira*. The thermodynamic and kinematic parameters are associated in Figure 4A, showing the tendency that pathogenic species are biased to the crawling state and can migrate longer distance on the host tissue surfaces. The crawling motility of *Leptospira* is caused by the attachment of the spirochete cell body to surfaces via adhesive cell surface components (Charon et al., 1981; Tahara et al., 2018). The successive alternation in the attachment and detachment of adhesins allows for this progressive movement by the spirochetes, however, an excessively strong adhesion can inhibit crawling (Tahara et al., 2018). LPS, the molecular basis for the identification of the different *Leptospira* serovars (Cerqueira and Picardeau, 2009; Picardeau, 2017), is thought to be a crucial adhesin important for this crawling motion (Tahara et al., 2018). Thus, it is possible that compatibility of the serological characteristics of leptospires and the surface properties of the host tissue might affect the crawling behavior over the tissue surfaces and the subsequent clinical consequences. The results of our biophysical experiments outline a plausible framework for the adhesion and crawling-dependent pathogenicity of *Leptospira* (Figure 4B). The biased transition from the adhesion to the crawling state and the long-distance, persistent crawling allows leptospires to explore the host’s cell surfaces, increasing the probability of encountering routes for invasion through their intracellular tight junctions (Figure 4B, left). In contrast, the swimming or weakly attached leptospires can be swept by external forces, such as intermittent urination. Furthermore, since leptospires cannot be disseminated over host tissues by diffusive crawling, such strains have less chance to discover routes for invasion (Figure 4B, right).

Other than LPS, abundant leptospiral proteins that are exposed to the cell exterior are known to possess the binding ability against extracellular matrix (ECM) components, such as fibronectin, laminin, and collagens (Haake and Matsunaga, 2010; Picardeau, 2017). For example, Lig (leptospiral immunoglobulin-like) proteins (Matsunaga et al., 2003) mediate the *Leptospira* adhesion via binding to fibronectin, laminin, collagen I, and collagen IV (Choy et al., 2007). LenA (leptospiral endostatin-like protein A) (Stevenson et al., 2007) that was formally called LfhA (leptospiral factor H-binding protein A)/Lsa24 (leptospiral surface adhesin 24) have binding property to the complement regulatory protein factor H (Verma et al., 2006) and laminin (Barbosa et al., 2006). OmpL1 serves as an adhesin through binding to laminin and plasma fibronectin (Fernandes et al., 2012; Robbins et al., 2015). Some of the adhesins are expressed only in pathogenic strains, suggesting that their combination could be involved in directive and persistent crawling observed in the pathogenic strains. Further study using other saprophytic and pathogenic *Leptospira* spp. is required for defining the detailed roles of these adhesive molecules for crawling. Also, *in vivo* experiments demonstrating direct correlation between crawling and virulence of *Leptospira* should be conducted.

Some bacterial pathogens are specialized to invade a very limited array of hosts, whereas others can infect multiple host species. The host range differs for each pathogen and the clinical symptoms depend on each host–pathogen combination. The same applies for leptospirosis, the outcome of *Leptospira* infection depends on the host-serovar association, and some animal species can become an asymptomatic reservoir for particular *Leptospira* serovars. The present experiments also provided data allowing us to discuss the host dependence of the leptospiral dynamics. Among the investigated materials, serovar Manilae vs. rats and serovar Icterohaemorrhagiae vs. rats are typically asymptomatic pairs, and Figure 4A shows that the pairs with reservoirs have lower scores in comparison with those causing severe symptoms, such as Manilae vs. humans, Icterohaemorrhagiae vs. dogs, and others. This implies that the surface dynamics of the spirochete could be related to their host-dependent pathogenicity. Understanding the mechanism of the host preferences by pathogens is important for prevention of the infection spread. There still remain gaps in our knowledge on the host–pathogen interaction in leptospirosis, but crucial insights into the issue have been provided by several research groups. Microarray analyses have revealed that regulation of gene expression in *Leptospira* is affected by its interaction with host cells (Toma et al., 2014; Satou et al., 2015). Although leptospiral LPS that is believed to be an adhesin for crawling induces the expression of proinflammatory cytokine genes through the recognition by the host TLR, the host-dependent difference in the TLR recognition was shown between human and murine cells: TLR1 and TLR2 predominantly mediate the activation in human cells, whereas TLR2 and TLR4 act for the recognition in mouse cells (Werts et al., 2001; Nahori et al., 2005). Pathogenic leptospires evade the host immunity through the interaction with the complement system (Barbosa and Isaac, 2020). Also, the binding of *L. interrogans* to thrombin inhibits fibrin coagulation, which results in hemorrhage (Fernandes et al., 2015). In relation to hemorrhage by *L. interrogans*, the pathogens acquire iron, that is believed to be essential during infection, from host erythrocyte through hemolytic activity of sphingomyelinases-like proteins (Sph) (Narayanavari et al., 2015). Thus, various *Leptospira*-host interactions such as immune evasion and nutrition acquisition could inclusively determine the host preference of *Leptospira*. Such abundant factors in both bacteria and hosts should be investigated for a deeper understanding of the host-dependent pathogenicity.

## Data Availability Statement

All datasets presented in this study are included in the article/Supplementary Material.

## Author Contributions

JX, NK, and SN planned the project and wrote the manuscript. JX and NK carried out the experiments. SN set up the optical system and programs for data analysis. JX and SN analyzed the data. All authors contributed to the article and approved the submitted version.

## Conflict of Interest

The authors declare that the research was conducted in the absence of any commercial or financial relationships that could be construed as a potential conflict of interest.

## Supplementary Material

FIGURE S1 Effect of GFP expression on the *Leptospira* motility.

FIGURE S2 Explanation of MSD plot.

FIGURE S3 Histograms of the MSD slopes.

TABLE S1 Primer sequences used in this study.

MOVIE S1 Epi-fluorescent images of *L. interrogans* on the rat kidney cell.

MOVIE S2 Progressive, long-distance crawling of *L. interrogans* on the monkey kidney cells.

MOVIE S3 Crawling of *L. interrogans* with highly frequent reversal on the dog kidney cells.

## References

[B1] Adler B. de la Peña Moctezuma A. (2010). *Leptospira* and leptospirosis. *Vet. Microbiol.* 140 287–296. 10.1016/j.vetmic.2009.03.012 19345023

[B2] Barbosa A. S. Abreu P. A. E. Neves F. O. Atzingen M. V. Watanabe M. M. Vieira M. L. (2006). A newly identified Leptospiral adhesin mediates attachment to laminin. *Infect. Immun.* 74 6356–6364. 10.1128/IAI.00460-06 16954400 PMC1695492

[B3] Barbosa A. S. Isaac L. (2020). Strategies used by *Leptospira* spirochetes to evade the host complement system. *FEBS Lett.* 10.1002/1873-3468.13768 (in press). 32153015

[B4] Bauby H. Saint Girons I. Picardeau M. (2003). Construction and complementation of the first auxotrophic mutant in the spirochaete *Leptospira meyeri*. *Microbiology* 149 689–693. 10.1099/mic.0.26065-0 12634337

[B5] Bharti A. R. Nally J. E. Ricaldi J. N. Matthias M. A. Diaz M. M. Lovett M. A. (2003). Leptospirosis: a zoonotic disease of global importance. *Lancet Infect. Dis.* 3 757–771. 10.1016/s1473-3099(03)00830-2 14652202

[B6] Bono J. L. Elias A. F. Kupko J. J. Stevenson B. Tilly K. Rosa P. (2000). Efficient targeted mutagenesis in *Borrelia burgdorferi*. *J. Bacteriol.* 182 2445–2452. 10.1128/JB.182.9.2445-2452.2000 10762244 PMC111306

[B7] Cerqueira G. M. Picardeau M. (2009). A century of *Leptospira* strain typing. *Infect. Genet. Evol.* 9 760–768. 10.1016/j.meegid.2009.06.009 19540362

[B8] Charon N. W. Lawrence C. W. O’Brien S. (1981). Movement of antibody-coated latex beads attached to the spirochete *Leptospira interrogans*. *Proc. Natl. Acad. Sci. U.S.A.* 78 7166–7170. 10.1073/pnas.78.11.7166 6947280 PMC349217

[B9] Cheney C. P. Schad P. A. Formal S. B. Boedeker E. C. (1980). Species specificity of *in vitro Escherichia coli* adherence to host intestinal cell membranes and its correlation with *in vivo* colonization and infectivity. *Infect. Immun.* 28 1019–1027. 6995316 10.1128/iai.28.3.1019-1027.1980 PMC551052

[B10] Choy H. A. Kelley M. M. Chen T. L. Møller A. K. Matsunaga J. Haake D. A. (2007). Physiological osmotic induction of *Leptospira interrogans* adhesion: LigA and LigB bind extracellular matrix proteins and fibrinogen. *Infect. Immun.* 75 2441–2450. 10.1128/IAI.01635-06 17296754 PMC1865782

[B11] Clyne M. Ocroinin T. Suerbaum S. Josenhans C. Drumm B. (2000). Adherence of isogenic flagellum-negative mutants of *Helicobacter pylori* and *Helicobacter mustelae* to human and ferret gastric epithelial cells. *Infect. Immun.* 68 4335–4339. 10.1128/iai.68.7.4335-4339.2000 10858255 PMC101762

[B12] Cox P. J. Twigg G. I. (1974). Leptospiral motility. *Nature* 250 260–261. 10.1038/250260a0 4852658

[B13] Ebady R. Niddam A. F. Boczula A. E. Kim Y. R. Gupta N. Tang T. T. (2016). Biomechanics of *Borrelia burgdorferi* vascular interactions. *Cell Rep.* 16 2593–2604. 10.1016/j.celrep.2016.08.013 27568563 PMC5235898

[B14] Fernandes L. G. de Morais Z. M. Vasconcellos S. A. Nascimento A. L. T. O. (2015). *Leptospira interrogans* reduces fibrin clot formation by modulating human thrombin activity via exosite I. *Pathog. Dis.* 73:ftv001. 10.1093/femspd/ftv001 25834144

[B15] Fernandes L. G. V. Vieira M. L. Kirchgatter K. Alves I. J. Morais Z. M. de Vasconcellos S. A. (2012). OmpL1 is an extracellular matrix- and plasminogen-interacting protein of *Leptospira* spp. *Infect. Immun.* 80 3679–3692. 10.1128/IAI.00474-12 22802342 PMC3457549

[B16] Fujita R. Koizumi N. Sugiyama H. Tomizawa R. Sato R. Ohnishi M. (2015). Comparison of bacterial burden and cytokine gene expression in golden hamsters in early phase of infection with two different strains of *Leptospira interrogans*. *PLoS One* 10:e0132694. 10.1371/journal.pone.0132694 26146835 PMC4492770

[B17] Goldstein S. F. Charon N. W. (1990). Multiple-exposure photographic analysis of a motile spirochete. *Proc. Natl. Acad. Sci. U.S.A.* 87 4895–4899. 10.1073/pnas.87.13.4895 2367518 PMC54227

[B18] Haake D. A. Matsunaga J. (2010). *Leptospira*: a spirochaete with a hybrid outer membrane. *Mol. Microbiol.* 77 805–814. 10.1111/j.1365-2958.2010.07262.x 20598085 PMC2976823

[B19] Josenhans C. Suerbaum S. (2002). The role of motility as a virulence factor in bacteria. *Int. J. Med. Microbiol.* 291 605–614. 10.1078/1438-4221-00173 12008914

[B20] Koizumi N. Izumiya H. Mu J.-J. Arent Z. Okano S. Nakajima C. (2015). Multiple-locus variable-number tandem repeat analysis of *Leptospira interrogans* and *Leptospira borgpetersenii* isolated from small feral and wild mammals in East Asia. *Infect. Genet. Evol.* 36 434–440. 10.1016/j.meegid.2015.08.013 26296603

[B21] Koizumi N. Watanabe H. (2004). Leptospiral immunoglobulin-like proteins elicit protective immunity. *Vaccine* 22 1545–1552. 10.1016/j.vaccine.2003.10.007 15063580

[B22] Kusumi A. Sako Y. Yamamoto M. (1993). Confined lateral diffusion of membrane receptors as studied by single particle tracking (nanovid microscopy). Effects of calcium-induced differentiation in cultured epithelial cells. *Biophys. J.* 65 2021–2040. 10.1016/S0006-3495(93)81253-0 8298032 PMC1225938

[B23] Lambert A. Picardeau M. Haake D. A. Sermswan R. W. Srikram A. Adler B. (2012). FlaA proteins in *Leptospira interrogans* are essential for motility and virulence but are not required for formation of the flagellum sheath. *Infect. Immun.* 80 2019–2025. 10.1128/IAI.00131-12 22451522 PMC3370569

[B24] Matsunaga J. Barocchi M. A. Croda J. Young T. A. Sanchez Y. Siqueira I. (2003). Pathogenic *Leptospira* species express surface-exposed proteins belonging to the bacterial immunoglobulin superfamily. *Mol. Microbiol.* 49 929–946. 10.1046/j.1365-2958.2003.03619.x 12890019 PMC1237129

[B25] Miyahara S. Saito M. Kanemaru T. Villanueva S. Y. A. M. Gloriani N. G. Yoshida S. (2014). Destruction of the hepatocyte junction by intercellular invasion of *Leptospira* causes jaundice in a hamster model of Weil’s disease. *Int. J. Exp. Path.* 95 271–281. 10.1111/iep.12085 24945433 PMC4170969

[B26] Nahori M.-A. Fournié-Amazouz E. Que-Gewirth N. S. Balloy V. Chignard M. Raetz C. R. H. (2005). Differential TLR recognition of leptospiral lipid A and lipopolysaccharide in murine and human cells. *J. Immunol.* 175 6022–6031. 10.4049/jimmunol.175.9.6022 16237097

[B27] Nakamura S. Leshansky A. Magariyama Y. Namba K. Kudo S. (2014). Direct measurement of helical cell motion of the spirochete *Leptospira*. *Biophys. J.* 106 47–54. 10.1016/j.bpj.2013.11.1118 24411236 PMC3907252

[B28] Narayanavari S. A. Lourdault K. Sritharan M. Haake D. A. Matsunaga J. (2015). Role of *sph2* gene regulation in hemolytic and sphingomyelinase activities produced by *Leptospira interrogans*. *PLoS Negl. Trop. Dis.* 9:e0003952. 10.1371/journal.pntd.0003952 26274394 PMC4537108

[B29] Olsen J. E. Hoegh-Andersen K. H. Casadesús J. Rosenkrantz J. T. Chadfield M. S. Thomsen L. E. (2013). The role of flagella and chemotaxis genes in host pathogen interaction of the host adapted *Salmonella enterica* serovar Dublin compared to the broad host range serovar *S*.Typhimurium. *BMC Microbiol.* 13:67. 10.1186/1471-2180-13-67 23530934 PMC3621167

[B30] Picardeau M. (2008). Conjugative transfer between *Escherichia coli* and *Leptospira* spp. as a new genetic tool. *Appl. Environ. Microbiol.* 74 319–322. 10.1128/AEM.02172-07 17993560 PMC2223205

[B31] Picardeau M. (2017). Virulence of the zoonotic agent of leptospirosis: still terra incognita? *Nat. Rev. Micro* 15 297–307. 10.1038/nrmicro.2017.5 28260786

[B32] Robbins G. T. Hahn B. L. Evangelista K. V. Padmore L. Aranda P. S. Coburn J. (2015). Evaluation of cell binding activities of *Leptospira* ECM adhesins. *PLoS Negl. Trop. Dis.* 9:e0003712. 10.1371/journal.pntd.0003712 25875373 PMC4397020

[B33] Santos L. A. Adhikarla H. Yan X. Wang Z. Fouts D. E. Vinetz J. M. (2018). Genomic comparison among global isolates of *L. interrogans* serovars Copenhageni and Icterohaemorrhagiae identified natural genetic variation caused by an Indel. *Front. Cell Infect. Microbiol.* 8:193. 10.3389/fcimb.2018.00193 29971217 PMC6018220

[B34] Satou K. Shimoji M. Tamotsu H. Juan A. Ashimine N. Shinzato M. (2015). Complete genome sequences of low-passage virulent and high-passage avirulent variants of pathogenic *Leptospira interrogans* serovar Manilae strain UP-MMC-NIID, originally isolated from a patient with severe leptospirosis, determined using PacBio single-molecule real-time technology. *Genome Announc* 3:e00882-15. 10.1128/genomeA.00882-15 26272567 PMC4536678

[B35] Siitonen A. Nurminen M. (1992). Bacterial motility is a colonization factor in experimental urinary tract infection. *Infect. Immun.* 60 3918–3920. 10.1128/iai.60.9.3918-3920.1992 1379992 PMC257410

[B36] Slamti L. Picardeau M. (2012). Construction of a library of random mutants in the spirochete *Leptospira biflexa* using a *mariner* transposon. *Methods Mol. Biol.* 859 169–176. 10.1007/978-1-61779-603-6_9 22367871

[B37] Stevenson B. Choy H. A. Pinne M. Rotondi M. L. Miller M. C. DeMoll E. (2007). *Leptospira interrogans* endostatin-like outer membrane proteins bind host fibronectin, laminin and regulators of complement. *PLoS One* 2:e1188. 10.1371/journal.pone.0001188 18000555 PMC2063517

[B38] Tahara H. Takabe K. Sasaki Y. Kasuga K. Kawamoto A. Koizumi N. (2018). The mechanism of two-phase motility in the spirochete *Leptospira*: swimming and crawling. *Sci. Adv.* 4:eaar7975. 10.1126/sciadv.aar7975 29854948 PMC5976277

[B39] Toma C. Murray G. L. Nohara T. Mizuyama M. Koizumi N. Adler B. (2014). Leptospiral outer membrane protein LMB216 is involved in enhancement of phagocytic uptake by macrophages. *Cell. Microbiol.* 16 1366–1377. 10.1111/cmi.12296 24655538

[B40] Tomizawa R. Sugiyama H. Sato R. Ohnishi M. Koizumi N. (2017). Male-specific pulmonary hemorrhage and cytokine gene expression in golden hamster in early-phase *Leptospira interrogans* serovar Hebdomadis infection. *Microb. Pathog.* 111 33–40. 10.1016/j.micpath.2017.08.016 28811249

[B41] Verma A. Hellwage J. Artiushin S. Zipfel P. F. Kraiczy P. Timoney J. F. (2006). LfhA, a novel factor H-binding protein of *Leptospira interrogans*. *Infect. Immun.* 74 2659–2666. 10.1128/IAI.74.5.2659-2666.2006 16622202 PMC1459737

[B42] Vincent A. T. Schiettekatte O. Goarant C. Neela V. K. Bernet E. Thibeaux R. (2019). Revisiting the taxonomy and evolution of pathogenicity of the genus *Leptospira* through the prism of genomics. *PLoS Negl. Trop. Dis.* 13:e0007270. 10.1371/journal.pntd.0007270 31120895 PMC6532842

[B43] Werts C. Tapping R. I. Mathison J. C. Chuang T. H. Kravchenko V. Saint Girons I. (2001). Leptospiral lipopolysaccharide activates cells through a TLR2-dependent mechanism. *Nat. Immunol.* 2 346–352. 10.1038/86354 11276206

[B44] Wunder E. A. Figueira C. P. Benaroudj N. Hu B. Tong B. A. Trajtenberg F. (2016). A novel flagellar sheath protein, FcpA, determines filament coiling, translational motility and virulence for the *Leptospira* spirochete. *Mol. Microbiol.* 101 457–470. 10.1111/mmi.13403 27113476 PMC4979076

[B45] Xu J. Koizumi N. Nakamura S. (2020). Insight into motility-dependent pathogenicity of the zoonotic spirochete *Leptospira*. *bioRxiv* [Preprint]. 10.1101/2020.02.11.944587

[B46] Zhu W.-N. Huang L.-L. Zeng L.-B. Zhuang X.-R. Chen C.-Y. Wang Y.-Z. (2014). Isolation and characterization of two novel plasmids from pathogenic *Leptospira interrogans* serogroup Canicola serovar Canicola strain Gui44. *PLoS Negl. Trop. Dis.* 8:e3103. 10.1371/journal.pntd.0003103 25144555 PMC4140679

