# Corrigendum: Crawling Motility on the Host Tissue Surfaces Is Associated With the Pathogenicity of the Zoonotic Spirochete *Leptospira*

**DOI:** 10.3389/fmicb.2021.736406

**Published:** 2021-08-17

**Authors:** Jun Xu, Nobuo Koizumi, Shuichi Nakamura

**Affiliations:** ^1^Department of Animal Microbiology, Graduate School of Agricultural Science, Tohoku University, Sendai, Japan; ^2^Department of Bacteriology I, National Institute of Infectious Diseases, Tokyo, Japan; ^3^Department of Applied Physics, Graduate School of Engineering, Tohoku University, Sendai, Japan

**Keywords:** bacterial motility, pathogenicity, spirochete, leptospirosis, zoonosis, host–pathogen association, biophysics, optical microscopy

In the original article, there was a mistake in **Supplementary Table S1** as published.

In the original version, “TATCGATACCGTCGATCACTTGTACAGCTCATCCATG” was shown as the reverse primer of AcGFP, but “GCTGGCCGGCGTCGATCACTTGTACAGCTCATCCATG” is correct. The corrected Supplementary Table S1 appears below.

**Supplementary Table S1:** Primer sequences used in this study.

**Target**	**Primer sequence (5' → 3')**	
	**Forward**	**Reverse**
*rep-parB-parA* (pGui1)	TCGACGCCGGCCAGCGGTTACTTCATAGCATCTTGGTTC	GCTGGAGCTCCACCGGCTCGACTCTTACGGTGTGTTAG
pNKLiG1 (pCjSpLe94 derivative)	CGGTGGAGCTCCAGCTTTG	GCTGGCCGGCGTCGAAAAGTAAGCACCTGTTATTGC
*flgB* promoter	TATCGATACCGTCGACCCGAGCTTCAAGGAAGATTTCCTA	ATGGAAACCTCCCTCATTTA
AcGFP	GAGGGAGGTTTCCATATGACCATGATTACGCCAAGC	GCTGGCCGGCGTCGATCACTTGTACAGCTCATCCATG

The authors apologize for this error and state that this does not change the scientific conclusions of the article in any way. The original article has been updated.

## Publisher's Note

All claims expressed in this article are solely those of the authors and do not necessarily represent those of their affiliated organizations, or those of the publisher, the editors and the reviewers. Any product that may be evaluated in this article, or claim that may be made by its manufacturer, is not guaranteed or endorsed by the publisher.

