# Supplementary material for: Crawling Motility on the Host Tissue Surfaces Is Associated With the Pathogenicity of the Zoonotic Spirochete Leptospira
Source: Front Microbiol. 2020 Aug 5;11:1886. doi: 10.3389/fmicb.2020.01886 (PMC7419657; doi:10.3389/fmicb.2020.01886)
Supplement: FIGURE S1 — Effect of GFP expression on the Leptospira motility. [file Data_Sheet_1.pdf]

Supplementary Material

Title: Crawling motility on the host tissue surfaces is associated with the pathogenicity of the zoonotic spirochete *Leptospira*

Authors: Jun Xu, Nobuo Koizumi, Shuichi Nakamura

Correspondence to: naka@bp.apph.tohoku.ac.jp

This PDF file includes:

Supplementary Figure S1. Effect of GFP expression on the *Leptospira* motility.

Supplementary Figure S2. Explanation of MSD plot.

Supplementary Figure S3. Histograms of the MSD slopes

Supplementary Tables S1. Primer sequences used in this study **[corrected on July 2021]**

Other Supplementary Materials for this manuscript include the following:

Supplementary Movie S1. Epi-fluorescent images of *L. interrogans* on the rat kidney cell

Supplementary Movie S2. Progressive, long-distance crawling of *L. interrogans* on the monkey kidney cells

Supplementary Movie S3. Crawling of *L. interrogans* with highly frequent reversal on the dog kidney cells

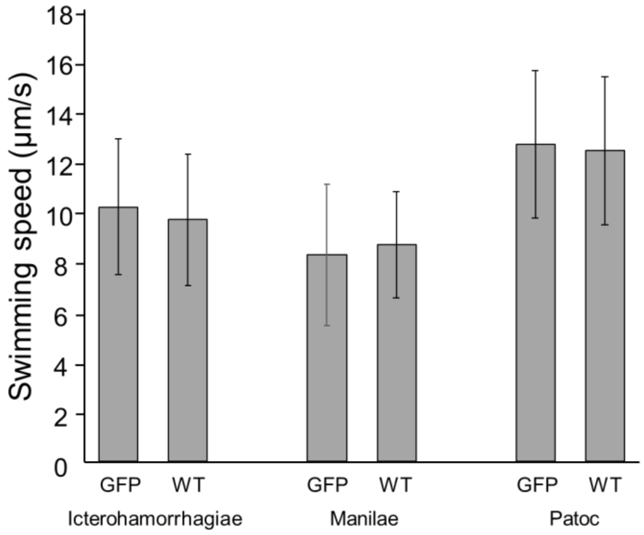

Supplementary Figure S1. Effect of GFP expression on the *Leptospira* motility. No significant difference was found between the swimming speeds of GFP expressing strains and wild type strains in each serovar.

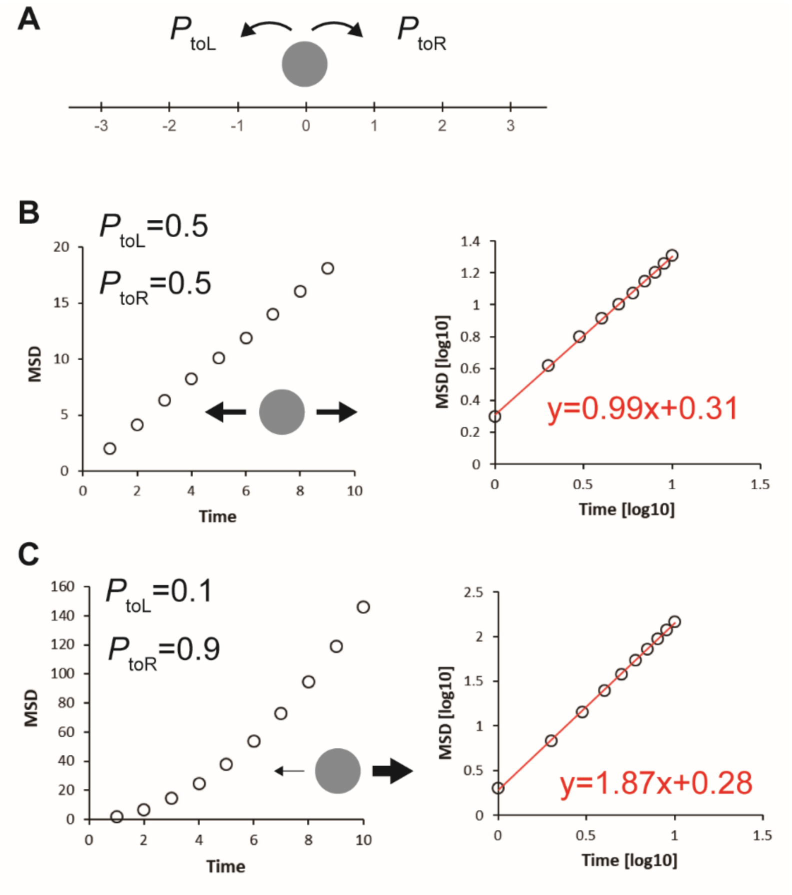

Supplementary Figure S2. Explanation of MSD plot. (A) To explain the difference in MSD vs time plot of "directed movement" (Figs. 3B-C upper) from that of "diffusive movement" (Figs. 3B-C lower), we performed a computer simulation by considering a particle stepping to the left (−1) or to the right (+1) with the probability of P_{toL} and P_{toR} (= 1−P_{toL}), respectively, in a time interval Δt. P_{toL} = 0.5 (P_{toR} = 0.5) and P_{toL} = 0.1 (P_{toR} = 0.9) were assumed for simulating simple diffusion and movement biased to the right, respectively, and the step direction was determined by a random number (*rnd*) from 0.0 to 1.0 generated in each event: If *rnd* < P_{toL}, the particle steps to the left (+1). The time course of the particle position was analyzed as shown in Methods. MSD vs time plots obtained by the simulation show that (B) simple diffusion and (C) directed movement give a linear line and a quadratic curve, respectively (left panels), therefore exhibiting linear lines with slopes of ~1 and ~2 in double-logarithmic plots (right panels). Red lines are regression lines fitted to data points obtained by simulation.

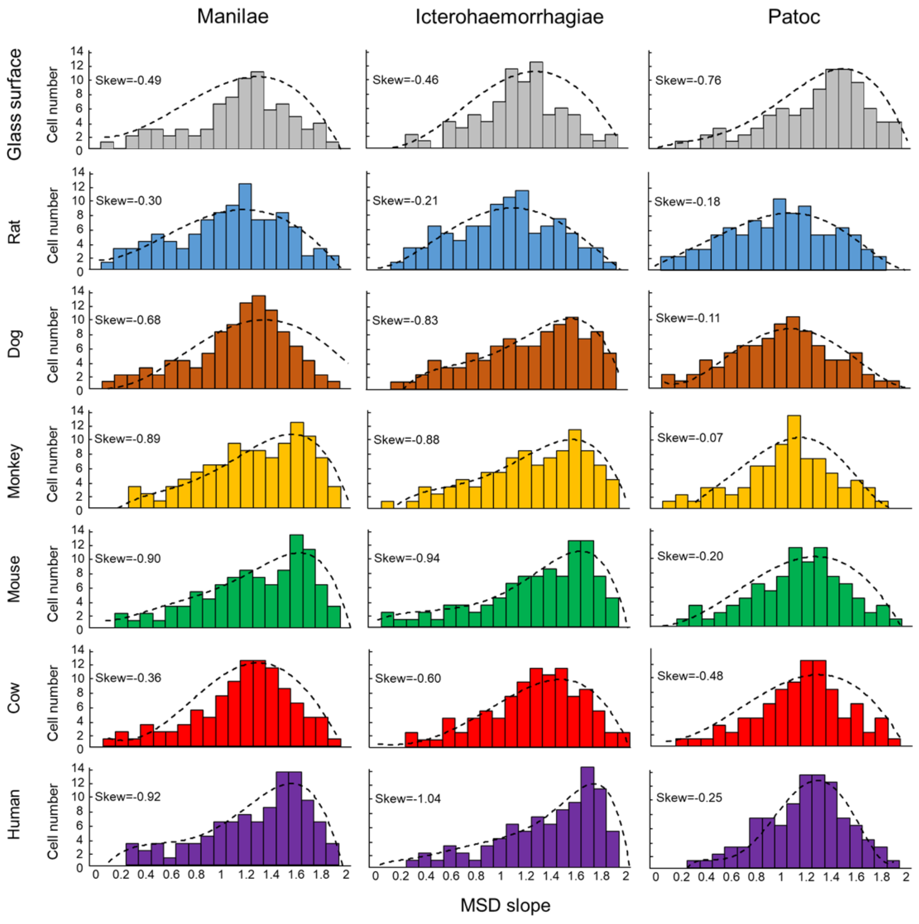

Supplementary Figure S3. Histograms of the MSD slopes. Dashed lines are the results of curve fitting.

Supplementary Table S1. Primer sequences used in this study [corrected on July 2021]

Target	Primer sequence (5´→ 3´)	
	Forward	Reverse
rep-parB-parA (pGui1)	TCGACGCCGGCCAGCGGTTACTTCATAGCATCTTGGTTC	GCTGGAGCTCCACCGGCTCGACTCTTACGGTGTGTTAG
pNKLiG1 (pCjSpLe94 derivative)	CGGTGGAGCTCCAGCTTTG	GCTGGCCGGCGTCGAAAAGTAAGCACCTGTTATTGC
flgB promoter	TATCGATACCGTCGACCCGAGCTTCAAGGAAGATTTCCTA	ATGGAAACCTCCCTCATTTA
AcGFP	GAGGGAGGTTTCCATATGACCATGATTACGCCAAGC	GCTGGCCGGCGTCGATCACTTGTACAGCTCATCCATG
